# Introduction to Special Issue Imaging in Cancer Diagnosis

**DOI:** 10.3390/tomography10010009

**Published:** 2024-01-15

**Authors:** Chiara Zanon, Emilio Quaia, Filippo Crimì

**Affiliations:** Institute of Radiology, Department of Medicine—DIMED, University of Padua, 35128 Padua, Italy; chiara.zanon@unipd.it (C.Z.); emilio.quaia@unipd.it (E.Q.)

In the field of oncology, the precision of cancer imaging is the cornerstone of oncological patient care. With the rapid advances in cancer therapies, there is a parallel need for specialized cancer imaging techniques [1].

This Special Issue aims to explore the role of new imaging technologies such as multi-parametric ultrasound (US), computed tomography (CT), Photon-counting CT (PCCT), magnetic resonance imaging (MRI) and positron emission tomography (PET) in cancer imaging.

US is playing an important role in the diagnosis of soft tissue tumors and in different abdominal neoplasms (e.g., prostate cancer (PCa), liver tumors, etc.), especially if aided by the use of US contrast medium intra-venous injection.

US is crucial in PCa diagnosis owing to advancements in elastography, contrast-enhanced techniques, and micro-ultrasound. These offer higher sensitivity, cost effectiveness, and real-time visualization, improving the accuracy of targeted biopsies and making it a more accessible alternative to traditional and MRI-based methods [2].

Additionally, US is essential for assessing soft tissue masses and fluid collections, providing a detailed first-line analysis of superficial anomalies. Despite challenges like distinguishing hematomas from sarcomas, US can aid in accurately identifying and understanding several soft tissue conditions [3].

Contrast-enhanced ultrasound (CEUS) is key for liver imaging; it provides detailed information for liver mass diagnosis and is particularly useful for distinguishing malignancies. It offers rapid, non-invasive diagnosis, enhances management of liver nodules, supports non-biopsy cancer identification, and visualizes tumor blood flow in real time, complementing CT and MRI in a comprehensive imaging strategy [4].

In the field of CT imaging, the recent introduction of PCCT aims to overcome the limitations of traditional CT detectors. PCCT not only enhances cardiovascular anatomy by providing better visuals of small arteries and stents, with less radiation exposure, but also is useful in oncological imaging [5,6].

The first studies published on PCCT in oncological imaging have shown a superiority of PCCT over conventional CT imaging in lung cancer screening (with higher sharpness, quality, and emphysema and nodule border clarity producing a lower radiation dose for the patients) and in detection of breast cancer primary lesions and metastases in a case series (with improved visualization of the lesion margins and content, along with less variability in size assessment) [7,8].

MRI is fundamental in oncological imaging because it provides more than just detailed anatomical information; it also offers functional and physiological data regarding the tumor microenvironment. Techniques such as perfusion MRI, spectroscopy and diffusion-weighted imaging (DWI) provide crucial information on angiogenesis, metabolism, cellularity, and hypoxia. These insights allow for earlier detection, better risk stratification, prognosis, and monitoring of treatment response, and they aid in new drug development, significantly enhancing cancer management [9].

Multiparametric MRI (mpMRI) significantly improves PCa diagnosis by reducing overdiagnoses and missed cases. It combines various imaging techniques (T1-weighted, T2-weighted, DWI, and dynamic contrast-enhanced imaging) for detailed assessment. mpMRI-targeted biopsies increase the detection of clinically significant cancers, particularly in men with prior negative or biopsy-naive biopsy results. While its role in reducing unnecessary biopsies is debated, mpMRI’s integration into PCa care is growing, despite ongoing challenges in quality, cost, and diagnostic integration [10].

MRI is also crucial for the diagnosis of hepatic lesions, distinguishing between malignant and benign lesions, and differentiating non-hepatocellular carcinoma (HCC) from HCC. It supports non-invasive HCC diagnosis in at-risk patients, as recommended by major medical guidelines. Despite challenges with atypical HCC features mimicking other malignancies, mpMRI options like hepatobiliary phase and DWI offer critical diagnostic information, aiding in accurate HCC diagnosis [11].

Additionally, hybrid imaging techniques, such as PET/CT and PET/MRI, which combine anatomical and metabolic data, have greatly advanced precision in staging and treatment strategies [12]. PET/CT is crucial to address patients toward palliative treatments or curative intent surgery due to its high accuracy in metastatic disease detection. Furthermore, PET/CT aids radiotherapy planning by incorporating tumor volumetric and radiobiological properties. These advancements have contributed to better patient outcomes, including survival rates, disease management, and quality of life [13]. A practical example could be the application of PET/CT in non-small cell lung cancer (NSCLC) where an accurate staging is the key for optimal patient management and the selection of treatment strategies. Over the past 20 years, FDG PET/CT has become a widespread imaging method for staging this cancer type, and is now routinely performed in major hospitals globally, providing essential information for clinical decision making [14].

PET/MRI has proven effective in assessing several cancers, including prostate, soft-tissue sarcoma, colorectal, and pediatric cancers, particularly when abdominal malignancy is suspected, despite negative results from other imaging methods [15].

Fluorine-18-labeled FDG PET/CT and MRI are crucial for the staging and assessment of pediatric extracranial solid tumors. PET/MRI merges these into one, providing both anatomic and physiologic insights in a single session, significantly reducing radiation exposure compared with CT. The advantages of PET/MRI include less radiation, fewer sedation and anesthesia events, and the combined use of two advanced imaging methods that are vital for evaluating treatment response in pediatric oncology [16].

PET/MRI combines the strengths of PET and MRI, allowing simultaneous image acquisition and precise image co-registration. MRI is becoming more prevalent for staging and restaging abdominopelvic cancers such as prostate, hepatobiliary, pancreatic, neuroendocrine, cervical, and rectal cancers. Fluorine 18-fluorodeoxyglucose PET/CT is a cornerstone of oncologic imaging, with the emergence of multiple targeted radiotracers that enhance its clinical use. Therefore, PET/MRI offers complementary imaging data, presenting clear advantages over separate PET/CT and MRI in terms of details and efficiency [17].

For example, PET/MRI is emerging as a potent tool for evaluating rectal cancer, offering detailed anatomical and functional insights while reducing radiation exposure compared to PET/CT. Its superior soft-tissue contrast helps in accurately determining local tumor size and, when combined with functional MRI techniques like DWI, enhancing patient care in oncology [18].

Another interesting field is the application of artificial intelligence (AI) in cancer assessment, including radiomics texture analysis and machine learning (ML). Radiomics Textural analysis (TA) uses raw data from various imaging techniques, such as US, CT, MRI, and PET. By examining pixel or voxel gray levels, the TA can detail cancer characteristics, assess heterogeneity, and predict pathological features, therapeutic responses, and prognoses [19,20,21].

The utilization of TA in medical imaging has markedly improved its capacity to distinguish between clear cell renal cell carcinoma (ccRCC) and non-ccRCC. Analysis of the texture features of renal tumors significantly improved the diagnostic accuracy and enhanced the differentiation between ccRCCs. This approach offers substantial improvements in predicting renal cancer types, crucial for tailored clinical treatments [20].

In the field of NSCLC, neural networks have proven to be powerful tools for correlating gene expression patterns with CT TA and distinct histological types. Neural networks have achieved high accuracy, histological classification, and radiomic feature prediction. Gene masking revealed associations between gene sets and radiomic features or histology types, with hypoxia and AKT signaling genes, showing strong predictive power [21].

CT TA in colorectal cancer (CRC) marks a significant advancement in non-invasive cancer diagnostics. The extraction of TA parameters from preoperative scans of patients with Stage III-IV CRC showed statistically significant differences in texture parameters between wild-type and mutated gene groups, indicating that CT TA can predict specific genetic mutations in CRC. This suggests a promising role for TA in noninvasive genetic assessments [22].

The synergy between AI and cancer imaging could further refine these cancer diagnostic processes, offering comprehensive insights into tumor biology by correlating radiomics with clinical, pathological, histological, and genetic data.

These are only a few of the new trends shaping cancer imaging in the future.

This Special Issue includes full research articles, case reports, and reviews focused on imaging in cancer diagnosis, showcasing a variety of innovative research across oncological imaging subdisciplines.
